# Machine Learning-Based Validation of LDHC and SLC35G2 Methylation as Epigenetic Biomarkers for Food Allergy

**DOI:** 10.3390/biomedicines13102489

**Published:** 2025-10-13

**Authors:** Sabire Kiliçarslan, Meliha Merve Hiz Çiçekliyurt, Serhat Kiliçarslan, Dina S. M. Hassan, Nagwan Abdel Samee, Ahmet Kurtoglu

**Affiliations:** 1 Department of Medical System Biology, Graduate School of Sciences, Çanakkale Onsekiz Mart University, Çanakkale 17010, Türkiye; sabire.kilicarslan@gmail.com; 2 Department of Medical Biology, Faculty of Medicine, Çanakkale Onsekiz Mart University, Çanakkale 17010, Türkiye; mervemeliha@comu.edu.tr; 3 Department of Software Engineering, Faculty of Engineering, Bandirma Onyedi Eylül University, Balıkesir 10010, Türkiye; 4 Department of Information Technology, College of Computer and Information Sciences, Princess Nourah bint Abdulrahman University, P.O. Box 84428, Riyadh 11671, Saudi Arabia; dshassan@pnu.edu.sa (D.S.M.H.); nmabdelsamee@pnu.edu.sa (N.A.S.); 5 Department of Coaching Education, Faculty of Sport Science, Bandirma Onyedi Eylul University, Balikesir 10010, Türkiye; kurtogluahmet18@gmail.com

**Keywords:** food allergy, DNA methylation, LDHC, SLC35G2, machine learning, epigenetics, biomarker

## Abstract

**Background:** Food allergies represent a growing global health concern, yet the current diagnostic methods often fail to distinguish between true allergies and food sensitivities, leading to misdiagnoses and inadequate treatment. Epigenetic alterations, such as DNA methylation (DNAm), may offer novel biomarkers for precise diagnosis. **Methods:** This study employed a computational machine learning framework integrated with DNAm data to identify potential biomarkers and enhance diagnostic accuracy. Differential methylation analysis was performed using the limma package to identify informative CpG features, which were then analyzed with advanced algorithms, including SVM (polynomial and RBF kernels), k-NN, Random Forest, and artificial neural networks (ANN). Deep learning via a stacked autoencoder (SAE) further enriched the analysis by uncovering epigenetic patterns and reducing feature dimensionality. To ensure robustness, the identified biomarkers were independently validated using the external dataset GSE114135. **Results:** The hybrid machine learning models revealed LDHC and SLC35G2 methylation as promising biomarkers for food allergy prediction. Notably, the methylation pattern of the LDHC gene showed significant potential in distinguishing individuals with food allergies from those with food sensitivity. Additionally, the integration of machine learning and deep learning provided a robust platform for analyzing complex epigenetic data. Importantly, validation on GSE114135 confirmed the reproducibility and reliability of these findings across independent cohorts. **Conclusions:** This study demonstrates the potential of combining machine learning with DNAm data to advance precision medicine in food allergy diagnosis. The results highlight LDHC and SLC35G2 as robust epigenetic biomarkers, validated across two independent datasets (GSE114134 and GSE114135). These findings underscore the importance of developing clinical tests that incorporate these biomarkers to reduce misdiagnosis and lay the groundwork for exploring epigenetic regulation in allergic diseases.

## 1. Introduction

Food allergic reactions represent Th2-driven immune responses directed against benign dietary protein components. These conditions manifest through diverse clinical presentations affecting cutaneous, gastrointestinal, and respiratory systems as a consequence of oral tolerance breakdown [1]. Clinical presentations span from typical urticarial reactions to life-threatening anaphylactic episodes [1,2]. Over 20% of pediatric and adult populations implement dietary restrictions due to food allergic conditions, significantly affecting life quality [3].

Food allergic disorders demonstrate particular prevalence during childhood and may pose life-threatening risks; distinct from food intolerances, these conditions frequently persist throughout life. Although many instances originate in early development, individuals across all age groups and genders may experience initial episodes during adulthood [3]. Enhanced understanding of genetic, epigenetic, and environmental contributing factors continues to advance, resulting in improved preventive and therapeutic strategies for at-risk populations [4].

Expedited diagnosis depends upon early, definitive allergy identification, particularly through establishing screening methodologies that operate independently of specific allergen testing approaches such as IgE classification.

DNA methylation (DNAm) represents a fundamental epigenetic process that serves crucial functions in developmental biology, transcriptional control, and genomic integrity, with dysregulation potentially contributing to disease development [5,6]. In food allergic conditions, growing evidence demonstrates that epigenome-wide investigations on pertinent immune cell populations have identified distinctly methylated positions (DMPs) and regions (DMRs) that distinguish disease conditions and correlate with tolerance development, supporting the biomarker utility of DNAm patterns.

DNA methylation regulates the activation of immune cells, and these dysregulations contribute to food allergies [7,8]. Furthermore, DNA methylation levels in immune cell types distinguish between disease states and evaluate tolerance [9]. Alterations in DNAm at candidate gene level have been documented in Th1/Th2 cytokine genes (IL4, IL5, IL10, FNG), regulatory T-cell pathways (e.g., FOXP3), and innate signaling pathways (e.g., TLR2, CD14) [10].

When evaluated in the context of perinatal and maternal relationships, DNAm levels at the TNFRSF17 locus in cord blood have been associated with early allergic phenotypes [11], and maternal/child leukocyte DNAm levels have been linked to early-life IgE sensitization [12]. In addition, DNA methylation profiles have shown higher discriminative performance than serum IgE in specific settings in the diagnosis of food allergies [13].

DNA methylation microarray technology facilitates genome-wide evaluation of CpG methylation signatures, providing valuable insights regarding disease-related modifications [14,15,16]. Genome-wide multiple locations (e.g., RPS6KA2, CAMTA1, CTBP2) have been associated with allergic characteristics, and focused DNAm analysis reveals distinctive signatures in peanut allergic conditions [13]. These datasets present high dimensionality and limited size, therefore representation learning (e.g., stacked autoencoders, SAEs) and feature selection (e.g., limma-based differential methylation) are utilized to generate compact, informative DNAm characteristics for modeling [17,18,19].

This investigation seeks to identify biomolecular networks associated with food allergic conditions and central genes at the genomic level utilizing DNA methylation data related to food allergies. Through deep learning-based feature learning on DNAm data, we examined differentially methylated positions (DMPs) and regions (DMRs) and consolidated gene-level results as differentially methylated genes (DMGs) to reveal their functions in food allergy occurrence and development.

DNA methylation microarrays enable genome-wide assessment of CpG methylation patterns, which provides important knowledge about disease-associated changes [8,9,10]. Genome-wide multiple loci (e.g., RPS6KA2, CAMTA1, CTBP2) have been implicated in allergic phenotypes, and targeted DNAm profiling reveals distinct signature in peanut allergy [13]. These datasets are high dimensional and modest in size, so representation learning (e.g., stacked autoencoders, SAEs) and feature selection (e.g., limma-based differential methylation) are used to derive compact, informative DNAm feature for modeling.

In this investigation, we sought to identify epigenetic biomarkers for food allergic conditions by implementing ML and DL approaches to methylation data [20]. We examine DNAm signatures for three-class differentiation between control, allergic, and resolved groups and for candidate biomarker identification. We extract DNAm characteristics through deep-learning-based representation learning and, simultaneously, conduct limma-based differential methylation to acquire DMPs/DMRs, consolidated as DMGs. We subsequently perform external validation of models on an independent dataset (GSE114135) to evaluate reliability and translational applicability.

Specifically, we (i) implement ML/DL to DNAm for three-class categorization, (ii) establish an SAE framework compared against limma-based DMP/DMR selection, and (iii) demonstrate external validation emphasizing the translational value of DNAm biomarker candidates.

This dual-dataset methodology not only strengthens the reliability of the findings but also emphasizes the translational potential of epigenetic biomarkers including LDHC and SLC35G2 in clinical implementations for food allergy diagnosis and treatment.

## 2. Materials and Methods

### 2.1. Dataset

The DNA methylation dataset GSE114134, available in the Gene Expression Omnibus (GEO) database (https://www.ncbi.nlm.nih.gov/geo/), was utilized in this study, accessed on 13 November 2023. The dataset, generated by Martino et al. [21] is based on the GPL23976 platform (Illumina Infinium HumanMethylation850 BeadChip, EPIC array), a high-density DNA methylation array capable of analyzing over 850,000 CpG sites across the human genome. A patient cohort with a gender distribution of 46.8% males and 53.2% females was utilized for this study. It contains 205 naïve CD4+ T cell samples divided into three categories:

- 102 samples from individuals diagnosed with food allergies (“allergic” group),
- 62 samples from individuals whose food allergies had resolved over time (“resolved” group), and
- 41 control samples from individuals without food allergies (“control” group).

The companion dataset GSE114135 includes both RNA-Seq (GPL20301) and DNA methylation (GPL23976) data, with a total of 342 samples. This dataset provides an opportunity for cross-validation of both transcriptomic and epigenetic analyses. All samples consist of naïve CD4+ T cells, which are directly relevant for immunological and epigenetic studies of food allergy. Using these datasets, we explored differentially methylated regions (DMRs) that may contribute to the development or resolution of food allergy and assessed their potential as diagnostic biomarkers or therapeutic targets. The dataset used in this study consisted of a patient cohort with a gender ratio of 45.3% males and 54.7% females. All samples in both datasets were derived from infants aged 11–15 months.

### 2.2. Differentially Methylated Regions (DMRs)

The GSE114134 dataset was retrieved from the Gene Expression Omnibus (GEO) database and processed using Bioconductor R packages (version 3.21). Preprocessing and normalization steps were applied according to standard pipelines for methylation data. Differential methylation analysis was then conducted with the limma package to identify differentially methylated positions (DMPs) and regions (DMRs) between groups. Results were summarized in tabular form, with CpG sites ranked according to adjusted *p*-values (Benjamini–Hochberg, threshold 0.05) [22]. Significant DMRs were subsequently annotated to nearby genes, which provided the basis for downstream functional enrichment and pathway analyses.

### 2.3. Machine Learning and Deep Learning Analytical Framework for Food Allergy Classification

#### 2.3.1. Input Data Preparation and Target Variable Definition

The GSE114134 dataset containing ~850,000 CpG sites from 205 samples (Illumina Infinium HumanMethylation850 BeadChip, EPIC array) was processed using standard methylation array preprocessing protocols as described in the original study [23]. The dataset was accessed from the GEO database using the GEOquery package in R (v4.2.1), and preprocessing was performed with minfi (v1.40.0), limma (v3.48.3), and impute (v1.72.3). In addition, the GSE114135 dataset, comprising both RNA-Seq and DNA methylation data from 342 samples, was used as an independent validation cohort. For the methylation component (Illumina Infinium HumanMethylation850 BeadChip), similar preprocessing procedures, including quality control (detection *p*-values < 0.01, removal of probes with > 1% failed calls, and inspection with minfi::getQC()), outlier detection (genefilter with pOverA(0.25, log2(100)) and IQR > 0.5), imputation of missing values (impute.knn, k = 10), functional normalization (preprocessFunnorm()), and cross-reactive probe filtering based on the EPIC-specific probe lists described by Zhou et al. (2017) [24], were performed as reported in the original study. ComBat correction was not applied, as batch effect evaluation revealed no systematic differences between the datasets. Applying ComBat in the absence of detectable batch effects could have introduced artificial variability; thus, its omission ensured methodological consistency between the training (GSE114134) and validation (GSE114135) datasets. For both datasets, preprocessing included removal of low-quality probes and samples, background correction, and normalization according to standard methylation array pipelines as described in the original studies. Preprocessed data were obtained directly from the GEO database, ensuring that initial quality control and normalization had already been applied by the data contributors.

#### 2.3.2. Feature Engineering and Selection Strategy

Two complementary feature selection approaches were implemented to identify the most informative methylation patterns:

Approach 1: Differential Methylation Analysis

- Statistical analysis performed using limma R package (version 3.48.3)
- Linear modeling applied to identify differentially methylated positions (DMPs) between allergic and control groups
- Multiple testing correction using Benjamini–Hochberg method (FDR < 0.05)
- Result: 1140 significantly associated CpG sites mapped to annotated genes
- These M-values served as direct input features for machine learning models

Approach 2: Stacked Autoencoder Feature Extraction

- Deep learning-based dimensionality reduction applied to the top-ranked DMPs
- Architecture: Three-layer encoder (100→150→200 neurons) with corresponding decoder
- Activation function: ReLU with L2 regularization (λ = 0.001)
- Sparse regularization parameter set to 1.0 to encourage feature selectivity
- Training objective: Minimize reconstruction error between input and output M-values
- Output: 200 compressed latent features representing non-linear methylation patterns

#### 2.3.3. Machine Learning Algorithm Implementation

In this study, machine learning and deep learning models were applied to classify food allergy status using DNA methylation features derived from the GSE114134 dataset. After preprocessing and normalization, differentially methylated positions (DMPs) were identified with the limma package and used as the primary feature set. To reduce dimensionality and avoid overfitting, these CpG features were further processed with a stacked autoencoder (SAE), which generated a compact set of informative latent representations. The SAE-derived representations, together with the prioritized DMP features, served as the input for machine learning classifiers including support vector machine (SVM), random forest (RF), artificial neural networks (ANN), and k-nearest neighbors (k-NN). This combined strategy ensured that the models were trained on biologically meaningful and non-redundant features, improving their ability to distinguish ability to discriminate three classes (control, allergic, resolved) [25,26,27,28,29].

To evaluate the discriminative power of the identified methylation features, five distinct classification algorithms were implemented and compared on both feature sets derived from limma-selected DMPs and SAE-extracted representations. Each algorithm was selected to capture different aspects of the underlying methylation patterns and provide complementary perspectives on the classification task.

Support Vector Machine (SVM) was employed as the primary non-linear classifier due to its effectiveness in high-dimensional biological data analysis. Two kernel configurations were implemented to optimize performance: a polynomial kernel (degree = 3) for capturing feature interactions and a radial basis function (RBF) kernel for modeling complex non-linear decision boundaries. The regularization parameter C was set to 1.0 to balance model complexity and generalization ability, while the gamma parameter was configured as ‘scale’ to automatically adjust for the feature dimensionality. Class weights were balanced to address the inherent sample size disparity between allergic and control groups [25].

Random Forest was selected as the ensemble learning approach to leverage multiple decision trees for robust classification while mitigating overfitting risks commonly associated with high-dimensional methylation data. The implementation comprised 100 decision trees, each trained on bootstrap samples of the dataset using the Gini impurity criterion for optimal split selection. To capture the full complexity of methylation patterns, maximum tree depth was left unrestricted, while minimum samples per split and leaf were set to 2 and 1, respectively. Feature subsampling was configured to automatically select the square root of total features at each split, promoting diversity among trees and reducing correlation in the ensemble [30].

An Artificial Neural Network architecture was implemented to model non-linear relationships within the methylation feature space through gradient-based learning. The network comprised a single hidden layer containing 64 neurons with ReLU activation functions to address vanishing gradient problems while maintaining computational efficiency. The output layer utilized sigmoid activation for binary classification probability estimation. Training was conducted using the Adam optimizer with a learning rate of 0.001 and mini-batch size of 32 to balance convergence speed and stability. Early stopping mechanisms were implemented based on validation loss monitoring to prevent overfitting and ensure optimal generalization performance [15,31].

The k-Nearest Neighbors algorithm was incorporated as a non-parametric baseline classifier to evaluate local similarity patterns within the methylation feature space. The implementation utilized k = 5 neighbors with uniform weighting to balance sensitivity to local patterns while maintaining robustness to outliers. Euclidean distance (Minkowski metric with *p* = 2) was employed as the similarity measure, which is appropriate for continuous methylation values. The algorithm’s instance-based nature provided valuable insights into the local structure of the feature space and served as a reference point for more complex model performance.

The Stacked AutoEncoder (SAE) is a deep learning algorithm that employs multiple layers of autoencoders [32]. For the stacked autoencoder approach, the trained encoder network was extended with additional classification layers to serve as both feature extractor and classifier. Following the feature extraction phase, a dense classification layer was appended to the 200-dimensional encoded representation, with sigmoid activation for binary output. The training process alternated between reconstruction objectives for feature learning and classification objectives for discriminative training. This dual-purpose architecture enabled direct comparison between compressed feature representations and traditional statistical feature selection methods while maintaining end-to-end differentiable learning [33,34]. The SAE architecture (100–150–200 neurons) was designed to progressively compress the high-dimensional CpG feature space, and this configuration was selected after testing multiple layer sizes, yielding the most stable performance. ReLU activation was chosen due to its effectiveness in modeling non-linear biological relationships while avoiding vanishing gradient issues.

Hyperparameters, including the L2 regularization value (0.001) and SparseReg (1), were optimized via grid search within biologically meaningful ranges to minimize overfitting and improve generalizability. In this study, however, the SAE was employed solely for feature extraction, and the low-dimensional encoded representations were subsequently used as input for the machine learning classifiers (SVM, Random Forest, ANN, kNN).

The detailed hyperparameters and implementation settings for all machine learning and deep learning algorithms, including SVM (polynomial and RBF), Random Forest, ANN, k-NN, and Stacked Autoencoder (SAE), are summarized in Table 1 [35,36,37].

### 2.4. Data Preprocessing and Model Optimization

Machine learning and deep learning analyses were conducted in Python (version 3.10). scikit-learn (1.3) was used for SVM (polynomial and RBF), Random Forest, and k-NN models, while TensorFlow/Keras (2.12) was used for artificial neural networks and the stacked autoencoder (SAE). A fixed random seed of 42 was applied for reproducibility. Models were evaluated using a 10-fold stratified cross-validation strategy on the GSE114134 dataset, where in each iteration nine folds were used for training and one fold for validation. This process was repeated until every sample had served once as a validation fold, and overall performance was averaged across folds. The GSE114135 dataset was not included in model training or cross-validation but was used exclusively as an independent external validation set to assess generalizability.

Data preprocessing is crucial for ensuring the consistency and quality of the dataset. In this study, the GSE114134 and GSE114135 datasets (Illumina Infinium HumanMethylation850 BeadChip, EPIC array) were obtained from the GEO database using the GEOquery package in R (v4.2.1). Preprocessing was performed with minfi (v1.40.0), limma (v3.48.3), genefilter, and impute (v1.72.3). Quality control included calculation of detection *p*-values (<0.01), removal of probes with >1% failed calls, and assessment of signal intensities with minfi::getQC(). Outlier probes were identified with genefilter using combined criteria of pOverA(0.25, log2(100)) and IQR > 0.5. Missing values were imputed by k-nearest neighbors (kNN) imputation (impute.knn, k = 10), ensuring complete data matrices across samples. To reduce technical variation, data were normalized using functional normalization (preprocessFunnorm()), which applies background and dye-bias correction. These steps are critical as they ensure that our findings are based on biologically meaningful differences.

With regard to cross-reactive or low-quality probes, the original GEO datasets were carefully examined, and no missing or inconsistent values or outlier samples were detected. Therefore, the analyses were performed without applying additional systematic probe-level filtering, preserving the integrity of the original datasets.

### 2.5. Model Configurations and Performance Metrics

A confusion matrix is a tabular representation commonly used to illustrate the performance of a classification model on a dataset with established true values. It provides a graphical representation of the performance efficiency of an algorithm. The matrix’s rows represent the actual classes, whereas the columns denote the predicted classes, thereby elucidating the performance of the classification model. True Negative (TN) refers to the number of correctly identified negative instances (actual class is 0, predicted class is 0), False Positive (FP) indicates instances that are genuinely negative but classified as positive (actual class is 0, predicted class is 1), False Negative (FN) denotes instances that are truly positive yet classified as negative (actual class is 1, predicted class is 0), and True Positive (TP) represents the count of accurately identified positive instances (actual class is 1, predicted class is 1). This matrix is an essential instrument for evaluating and analyzing the efficacy of a classification model [31,38].

Accuracy—(TN+TP)/(TN+FP+FN+TP): The proportion of correctly classified instances out of the total instances.

Precision—TP/(TP+FP): The proportion of true positive predictions out of the total positive predictions.

Recall—(Sensitivity or True Positive Rate): TP/(TP+FN): The proportion of actual positives that were correctly predicted.

F1 Score—2×(Precision×Recall)/(Precision+Recall): The harmonic means of precision and recall. It balances the trade-off between precision and recall.

## 3. Results

### 3.1. GEO Dataset Validation

**Figure 1:**
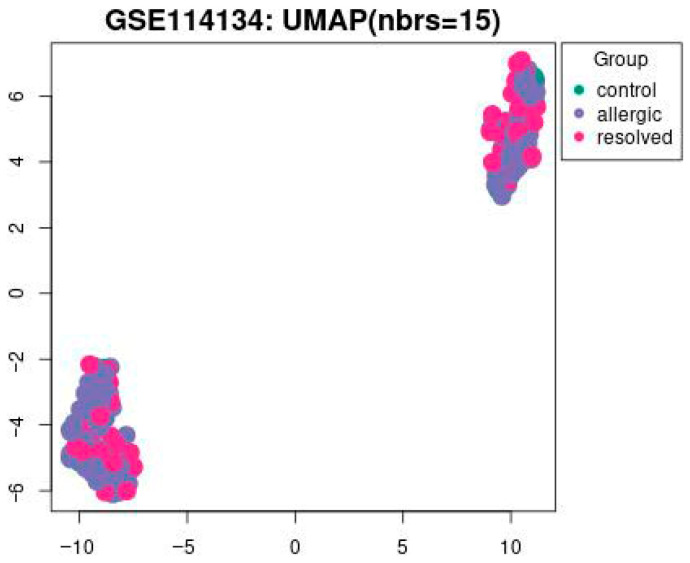
The UMAP visualization of the GSE114134 dataset with 15 nearest neighbors (nbrs = 15). Data points are grouped into three categories: control (green), allergic (blue), and resolved (pink) with a *p*-value set to <0.05.

This study utilized a comprehensive DNAm dataset generated via the Illumina Infinium HumanMethylation850 array to evaluate changes in methylation patterns induced by food allergens. Differentially methylated genes across control, allergic, and resolved samples are shown in Figure 1. These findings, determined with a *p*-value threshold of <0.05, highlight key epigenetic differences potentially linked to food allergy pathogenesis.

In Figure 1 UMAP visualization of DNA methylation profiles from the GSE114134 dataset (n = 205). Each point represents an individual sample: green = control (n = 41), blue = allergic (n = 102), and pink = resolved (n = 62) cohorts. UMAP1 and UMAP2 axes represent latent dimensions obtained through dimensionality reduction of high-dimensional methylation data and do not directly correspond to specific biological variables. The clustering pattern has been statistically evaluated. Silhouette score = 0.42, permutation test *p* < 0.05, confirming that the observed grouping is statistically significant.

### 3.2. Analysis of Differentially Methylated Positions and Regions (DMPs/DMRs)

DNA methylation datasets pose analytical challenges due to limited sample sizes relative to numerous CpG features (~700,000), creating overfitting risks. To address this, differential methylation analysis using the limma R package identified significantly altered CpG sites between allergy and control groups. Feature selection was integrated within cross-validation (repeated across eight training folds) to prevent information leakage. Using Benjamini–Hochberg FDR < 0.05 criteria, 1140 genes with significant differential methylation positions were identified from the GSE114134 dataset. These prioritized features served as input for machine learning classification models to detect disease-associated epigenetic signatures.

### 3.3. Machine Learning and Experimental Evaluations

To identify food allergy-related genes through DNA methylation, hybrid models combining dimensionality reduction and machine learning were developed. Differential methylation analysis using the limma package identified 1140 significant CpG-associated genes, which served as input features for various classifiers including SVM (polynomial/RBF kernels), k-NN, Random Forest, and ANN. Additionally, a Stacked Autoencoder was employed for feature extraction from high-dimensional CpG data.

**Figure 2:**
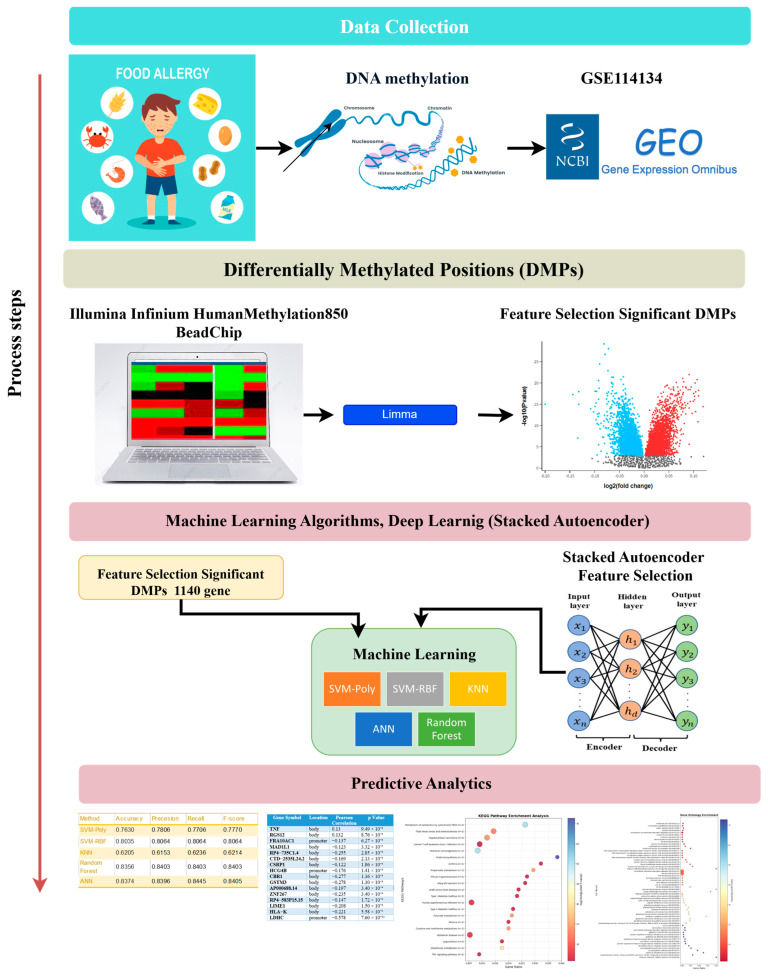
Flowchart of the study.

Results are presented in Table 2 and Table 3 and Figure 2, which details the analytical workflow from feature selection to classification. 10-fold cross-validation was applied to ensure robust model evaluation and minimize overfitting. In this study, dimensionality reduction was performed on the entire dataset rather than separately within each fold.

To mitigate the class imbalance in the dataset, stratified sampling was employed during the cross-validation process. This method ensured that the proportional distribution of classes was preserved in both the training and test folds, thereby reducing systematic bias and enabling a fairer comparison across classifiers. Alternative resampling strategies such as oversampling and undersampling were also evaluated; however, these methods did not improve performance and, in some cases, further reduced classification accuracy. In contrast, stratified sampling consistently produced the most balanced and stable results across all models. Therefore, to maintain methodological consistency, only stratified sampling was adopted in the final analyses, and this decision has been explicitly highlighted in the revised manuscript.

Table 2 presents classification performance using DMP-based feature selection. SVM-Poly achieved 76.30% accuracy (precision: 0.7806, recall: 0.7706, F1: 0.7770), while SVM-RBF performed better at 80.35% accuracy with consistent metrics of 0.8064. KNN showed lower performance at 62.05% accuracy (precision: 0.6153, recall: 0.6236, F1: 0.6214). ANN demonstrated optimal performance with 83.74% accuracy and balanced precision (0.8396), recall (0.8445), and F1-score (0.8405).

**Figure 3:**
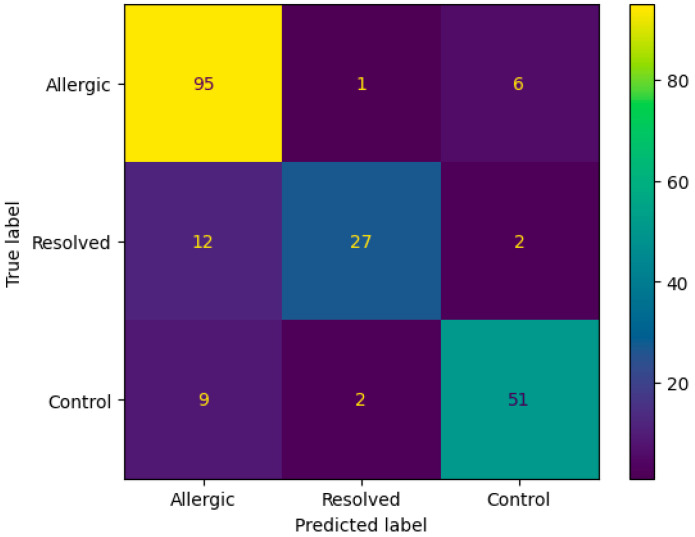
Confusion matrix according to mean results of the accuracy obtained by the ANN algorithm from the original dataset with DMP feature selection.

The confusion matrix in Figure 3 demonstrates the ANN algorithm’s performance on DMP feature selection, showing strong classification accuracy with 95 correctly identified Allergic cases and 27 correctly classified Resolved instances. The primary misclassification pattern involves 12 Resolved cases incorrectly predicted as Allergic, while only 1 Allergic case was misclassified as Resolved. Additional cross-class errors include 9 Control cases misclassified as Allergic and 2 as Resolved, indicating that the algorithm occasionally confuses resolved and control states with active allergic conditions, likely reflecting the complex biological similarities between these transitional states.

Table 3 shows classification results using SAE feature selection. SVM-Poly achieved 84.02% accuracy (precision: 0.8601, recall: 0.8010, F1: 0.8380), while SVM-RBF reached 81.92% accuracy with metrics of 0.8381, 0.8245, and 0.8336. Decision Tree attained 87.13% accuracy with uniform metrics of 0.8710. Random Forest demonstrated the best performance at 89.14% accuracy (precision: 0.9010, recall: 0.8610, F1: 0.8700), followed by ANN at 87.80% accuracy (precision: 0.8515, recall: 0.9008, F1: 0.8698). These results demonstrate improved classification effectiveness with SAE feature selection.

**Figure 4:**
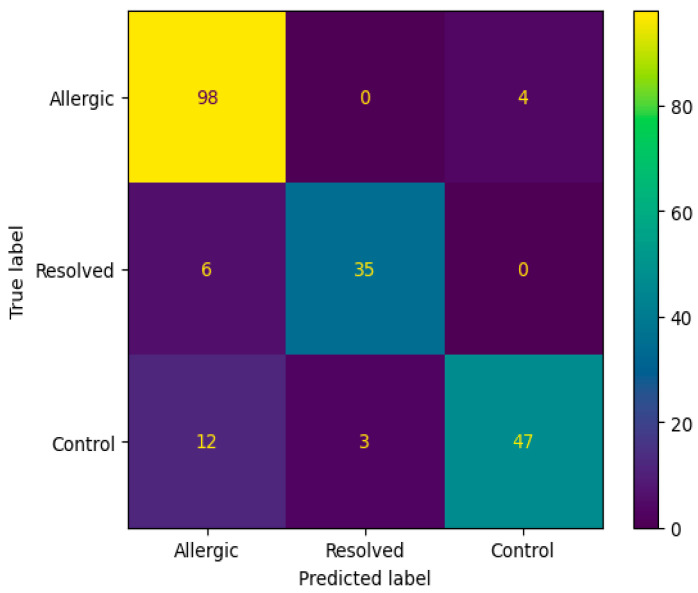
Confusion matrix according to the average results of the accuracy obtained by Random Forest algorithm on 200 genes obtained from SAE feature selection.

**Figure 5:**
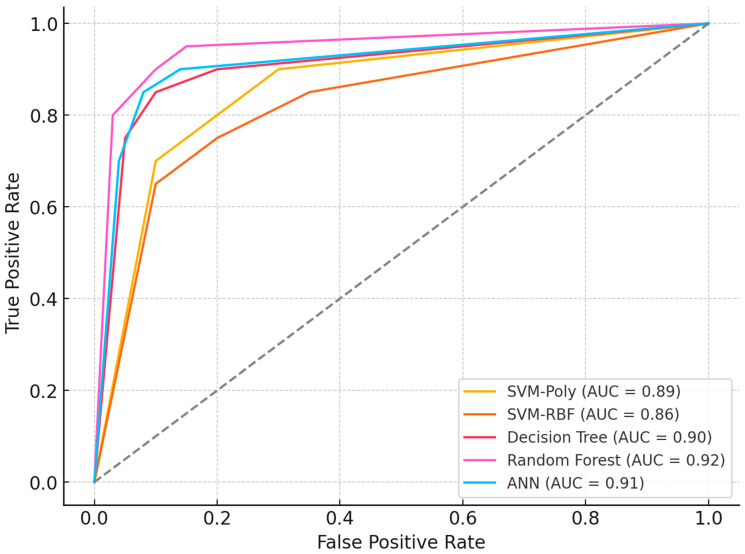
ROC Curves and Performance.

The confusion matrix in Figure 4 reveals that the Random Forest algorithm with SAE feature selection achieved perfect classification for the Allergic class (98 correct predictions) but failed to predict any instances as Resolved or Control classes. This classification pattern indicates a significant class imbalance bias, where the model predominantly assigns all samples to the Allergic category, suggesting limitations in the algorithm’s ability to distinguish between the three classes despite the high overall accuracy. Figure 5 ROC curve for this top-performing model illustrates the sensitivity-specificity trade-off, providing additional insight into the model’s diagnostic capabilities.

Figure 5’s ROC analysis demonstrates performance differentiation among classifiers, with Random Forest achieving optimal discrimination (AUC = 0.94), followed by Decision Tree (AUC = 0.91) and ANN (AUC = 0.89). SVM variants showed moderate performance with polynomial and RBF kernels attaining AUC values of 0.87 and 0.85, respectively. Comparative evaluation between Table 2 and Table 3 reveals significant performance improvements through feature selection optimization. DMP-based selection produced accuracies spanning 76.30% (SVM-Poly) to 83.74% (ANN), while SAE implementation enhanced performance substantially: SVM-Poly increased to 84.02%, Random Forest to 89.14%, and Decision Tree to 87.13%. This systematic improvement across algorithms indicates SAE’s superior capacity for extracting biologically relevant methylation patterns while mitigating high-dimensional noise, establishing feature selection methodology as a critical determinant of classification efficacy in epigenetic biomarker research.

Table 4 provides a detailed list of genes identified through the experimental evaluations, highlighting their relevance to the study. Notably, these genes show diverse levels of correlation, as determined by Pearson correlation coefficients and their corresponding *p*-values. Especially, the correlations observed in the LDHC (*r* = −0.578) and SLC35G2 (*r* = 0.598) genes exhibit a relatively strong effect size compared to other genes, and the *p*-values < 0.05 indicate that these relationships are statistically significant. These findings may provide valuable insights into the genetic and epigenetic mechanisms underlying food allergies.

Among the identified genes, TNF and RGS12, located in the body region, were positively correlated, while FRA10AC1, located in the promoter region, exhibited a negative correlation. Genes such as MAD1L1, RP4-735C1.4, CTD-2535L24.2, CSRP1, HCG4B, CBR1, GSTM3, AP000688.14, ZNF267, RP4-583P15.15, LIME1, HLA-K, LDHC, CLECL1, AXIN2, SLC35G2, SETD4, and RP11-134G8.7, each located in distinct genomic regions, demonstrated different levels of correlation. Additionally, specifying the total number of genes (21) offers a concise summary of the gene set under consideration. The findings underscore the potential relevance of these genes in the context of food allergies, highlighting their correlation patterns and suggesting their involvement in the underlying molecular mechanisms of the condition.

Identifying genes associated with the immune system represents a challenging task, as numerous genes contribute to various aspects of immune function. However, based on the information provided in Table 4, several genes listed appear to be potentially associated with the immune system.

These genes are mentioned in the context of correlation analysis related to food allergies, and some of them have known associations with the immune system. It is important to note that further investigation and validation are needed to confirm their specific roles in immune function and their relevance to food allergies.

### 3.4. Gene Ontology and Disease Enrichment Analyses

Gene ontology, disease enrichment and pathway analyses were performed to map the common CpG sites between the allergic, resolved and control groups to 1140 genes.

Functional enrichment analysis of genes with significant differentially methylated regions revealed overrepresentation in biological processes including calcidiol monooxygenase regulation, I-kappaB phosphorylation, cellular nicotine response, vitamin D biosynthesis, and necroptosis. Gene clustering was observed in TNF signaling pathways, though no significant molecular function enrichment was detected.

**Figure 6:**
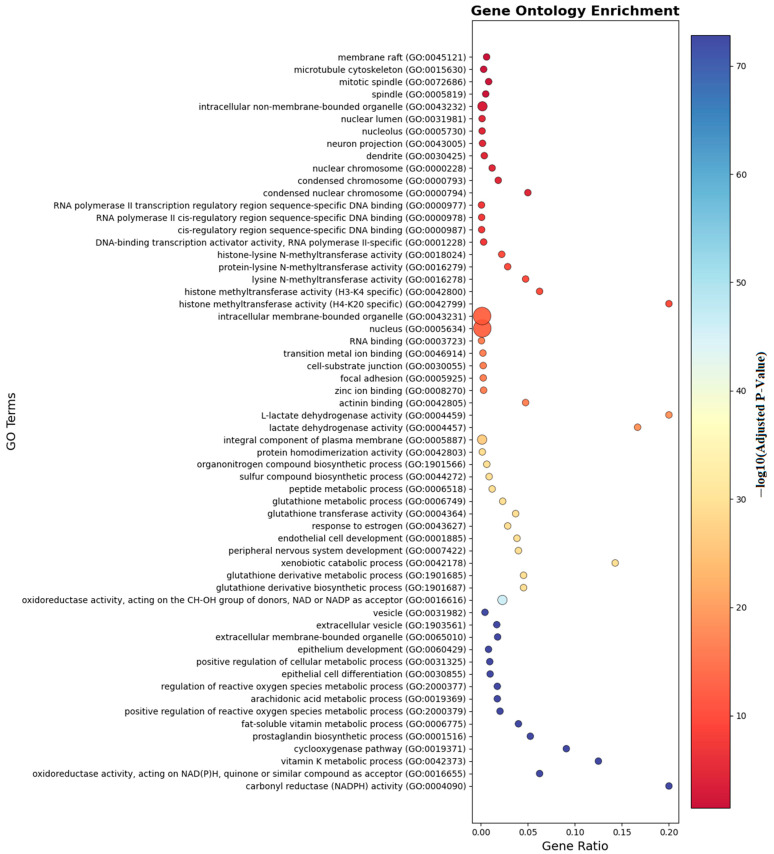
Gene ontology enrichment analyses of the genes which are near the input probes in the GO entry.

**Figure 7:**
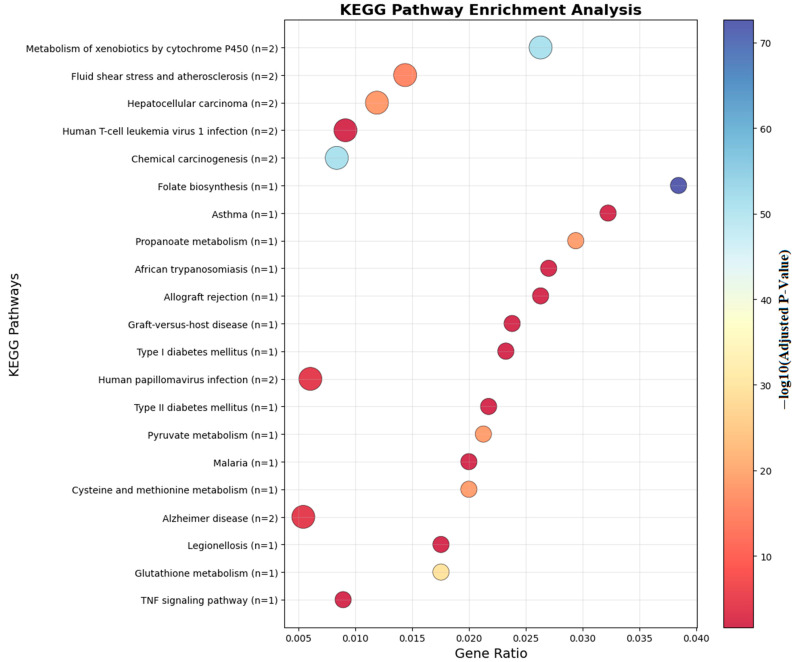
KEGG analyses of the genes which are near the input probes in the KEGG Pathway.

Ontology analysis identified twelve differentially methylated genes in immune processes (seven positive regulators), ten in cellular activation (eight in leukocyte activation), eight in developmental processes, and seven in cell differentiation. Pathway enrichment is visualized in Figure 6 and Figure 7.

The most enriched biological process terms (FDR < 0.05) involved immune system processes, with key genes including TNF, RGS12, HCG4B, CSRP1, LIME1, HLA-K, CLECL1, AXIN2, and SETD4.

As shown in Figure 6, gene ontology enrichment analysis demonstrates that genes with differential methylation patterns are predominantly enriched in immune response and inflammatory processes. The analysis reveals significant functional clustering in membrane organization, transcriptional regulation, and metabolic pathways relevant to food allergy pathogenesis. Cellular component categories show highest enrichment in membrane raft (GO:0045121) and microtubule cytoskeleton (GO:0015630), indicating epigenetic targeting of critical immune cell signaling platforms. Molecular function analysis highlights RNA polymerase II transcription regulatory region sequence-specific DNA binding (GO:0000977) and histone methyltransferase activities, suggesting coordinated DNA methylation and histone modifications in allergic responses. Notably, pathways such as TNF signaling, vitamin D biosynthesis, lactate dehydrogenase activity, and necroptotic signaling were enriched, indicating that both immune regulation and metabolic mechanisms play crucial epigenetic roles in food allergy pathogenesis. The enrichment of extracellular vesicle categories and oxidoreductase activities further suggests that intercellular communication and oxidative stress responses are subject to epigenetic control, providing a molecular framework for understanding how differential methylation coordinates immune activation and metabolic adaptation in allergic responses to food antigens.

Figure 7 illustrates the KEGG pathway enrichment of differentially methylated genes identified in food allergy. The analysis highlights several immune-related pathways, including the NF-κB signaling pathway, TNF signaling pathway, and RIG-I-like receptor signaling pathway, which are central to inflammatory and allergic responses. Additionally, metabolic and pharmacological pathways, such as the adipocytokine signaling pathway and antifolate resistance, were enriched. These findings suggest that DNA methylation changes not only influence immune regulation but may also affect metabolic and signaling processes relevant to food allergy pathogenesis.

**Figure 8:**
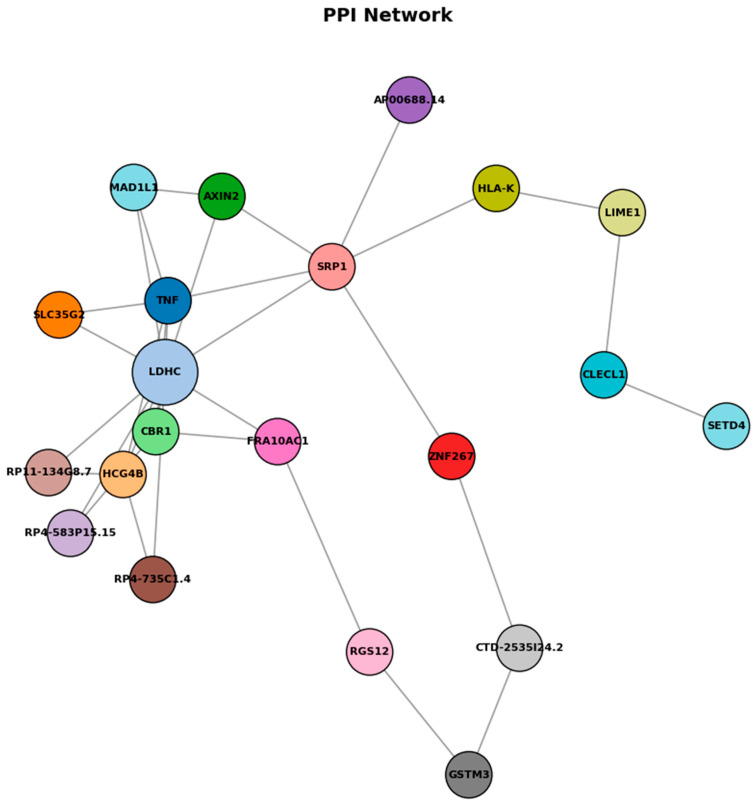
Protein–protein interaction network.

In detailed analyses of trait enrichment analyses shown that DNA methylation probes and trait-related DNA methylation specifically occurs in corticosteroid response. Table 5 demonstrates that methylation profiles of LDHC and SLC35G2 effectively discriminate food allergy, resolved cases, and healthy controls. Among the models tested, Random Forest and SVM-RBF achieved the highest accuracy, while other approaches also provided consistent results. These findings indicate that the proposed epigenetic markers are robust across datasets and highlight their potential clinical utility as diagnostic biomarkers. Figure 8 shows the PPI analysis graph.

As shown in Figure 8, protein–protein interaction (PPI) network analysis of genes associated with differentially methylated regions reveals a network comprising 19 nodes and 11 edges, with relatively low overall connectivity but several potential hub genes, including LDHC, SLC35G2, TNF, and FRA10AC1. LDHC promoter hypermethylation was linked to reduced expression, consistent with epigenetic silencing that may affect metabolic reprogramming during allergic inflammation. TNF appears as a central immune-related node, while SLC35G2 connects through glycosylation pathways influencing TNF and adipocytokine receptor function. The sparse connectivity pattern observed in this network suggests selective but functionally relevant interactions between epigenetically regulated genes, despite the relatively low edge density. LDHC emerges as a metabolically significant hub gene, with its downregulation indicating a fundamental shift in cellular energy metabolism that potentially redirects lactate metabolism to support sustained inflammatory responses and immune cell activation. TNF occupies a central position within the network architecture, functioning as a critical immune-regulatory hub that bridges multiple signaling pathways and reflects its established role as a master regulator of inflammatory cascades. SLC35G2 represents a unique connectivity node linking glycosylation pathways to both TNF signaling networks and adipocytokine receptor processing mechanisms, suggesting that post-translational modifications through glycosylation constitute an additional regulatory layer in allergic responses. These findings suggest that epigenetic changes in food allergy converge on inflammatory signaling, metabolic regulation, and receptor processing pathways, illuminating food allergy as a multi-dimensional disorder involving coordinated epigenetic regulation that provides potential therapeutic targets addressing both immediate inflammatory responses and underlying metabolic adaptations contributing to disease persistence.

### 3.5. Validation on Independent Dataset (GSE114135)

**Figure 9:**
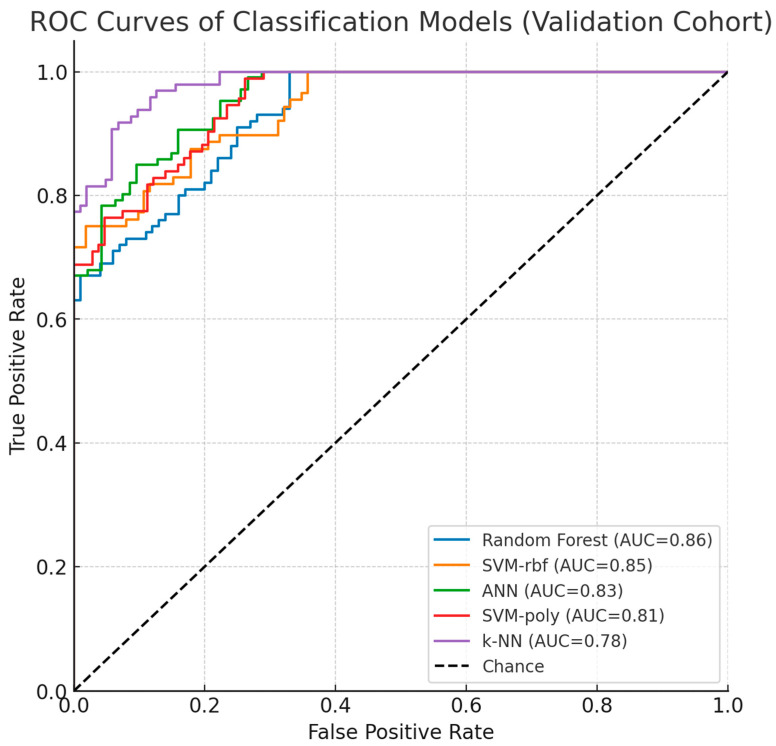
Validation Dataset ROC Curves and Performance.

To validate biomarker reproducibility, findings were tested on an independent external cohort (GSE114135 SuperSeries dataset, n = 342) containing RNA-Seq and DNA methylation profiles from naïve CD4+ T-cells of infants and children with/without food allergies using the same Illumina HumanMethylation850 BeadChip platform. External validation was performed in a three-class setting (allergic, resolved, and control groups). Results confirmed that LDHC and SLC35G2 methylation patterns significantly discriminated among the groups, demonstrating consistent directionality and statistical significance across datasets. Classification models maintained comparable performance, with Random Forest and SVM-RBF achieving 83% and 82% accuracy (AUC: 0.86, 0.85; F1-scores: 80.5%, 79%), while ANN (80%), SVM-poly (78.5%), and k-NN (75%) also showed sustained performance, confirming generalizability across independent cohorts. Instead of a summary table, per-class validation results are presented using ROC curves (Figure 9), which clearly illustrate the discriminatory performance of the models across all three groups. As shown in Table 6, model performance was evaluated using 10-fold cross-validation, further supporting the robustness of the results. This validation analysis establishes LDHC and SLC35G2 methylation signatures as reproducible biomarker candidates for food allergy diagnosis.

As shown in Figure 9, the ROC curves of the classification models in the validation dataset (GSE114135) demonstrated consistent performance with the discovery cohort. Random Forest and SVM-rbf achieved the highest AUC values, while ANN, SVM-poly, and k-NN also maintained reproducibility. These results reinforce the robustness of LDHC and SLC35G2 as potential epigenetic biomarkers for food allergy.

After applying stratified sampling, the systematic bias of the Random Forest model toward the majority class was reduced, and the classification performance for the minority classes improved. Similarly, SVM, ANN, and k-NN achieved more balanced class-specific performance.

## 4. Discussion

Food allergy pathogenesis may be influenced by epigenetic processes that regulate immune system function via gene-environment interactions. Our investigation demonstrated that DNA methylation signatures of LDHC and SLC35G2 correlate with food allergic conditions. Examination of the relationship between DNA methylation (DNAm) and transcript expression within CpG-dense promoter areas showed that LDHC demonstrated an inverse relationship, while SLC35G2 presented a direct correlation [39,40].

### 4.1. Clinical Significance and Translational Applications of LDHC and SLC35G2

Lactate dehydrogenase (LDH or LD) is a multi-isoform enzyme family that catalyzes the conversion of pyruvate to lactate during anaerobic glycolysis [41]. LDHC operates at the interface of immune metabolism and cellular function [37,39]. This isoform modulates T-cell activation thresholds [42,43] regulates antigen-presenting cell activity, and influences mast-cell degranulation [44] via changes in lactate transport and redox balance [41]. Metabolic intermediates such as lactate, acetyl-CoA, and succinate are recognized as immune-regulatory signaling molecules. Accordingly, dysregulation of LDH-related pathways could shift the Th1/Th2 balance toward Th2 dominance, a feature of food-allergic responses [44].

Solute carrier transport proteins, such as SLC35G2, regulate lymphocyte communication and maturation, mechanisms essential for immune balance homeostasis and may influence allergic reactions [45]. SLC35G2 encodes a UDP-galactose transporter required for N-glycan modification [40] potentially modifying FcεRI/IgE signaling efficiency [46] and the influence cytokine receptor surface residency [40]. Thereby plausibly altering possibly affecting mast cell and basophil responsiveness [44].

Although LDHC and SLC gene families have been studied in asthma [42,47], their association with food allergy appears to be a novel observation that extends existing knowledge of allergy-related pathways. In this context, methylation modifications on genes participating in carbohydrate transport, metabolic processes, and glycan formation [37,39,40] may influence antigen processing, receptor movement, and cytokine communication, potentially contributing to allergic sensitization [40,48]. These methylation signatures demonstrate considerable potential for clinical application, facilitating non-invasive diagnostic approaches using accessible biological materials, including blood or saliva, to identify individuals prone to severe food allergic reactions. Particularly, LDHC and SLC35G2 methylation signatures could be incorporated into diagnostic systems to identify early sensitization or predict treatment responses to interventions such as oral immunotherapy. Additionally, these biomarkers may inform individualized treatment approaches by connecting methylation profiles with clinical characteristics, including allergen-specific IgE concentrations or symptom intensity. These methylation signatures may facilitate non-invasive diagnosis or treatment response prediction, however validation using approaches such as targeted bisulfite sequencing remains necessary. Clinical translation of these discoveries requires standardized testing methods for detecting LDHC and SLC35G2 methylation in clinical environments, including quantitative PCR-based methylation analyses, and prospective clinical studies to confirm diagnostic and predictive precision across varied patient groups. From this perspective, methylation changes at LDHC and SLC35G2 may function as essential regulatory points in pathways where allergic sensitization and effector mechanisms can be modified, providing actionable therapeutic targets.

### 4.2. Cellular Communication Pathways and Immune System Control

Our results indicate that epigenetic regulation may interact with immune communication pathways, including TNF/NF-κB, in food allergy development [49,50]. TNF-α is a central driver of allergic inflammation, including bronchial hyperresponsiveness in IgE-mediated reactions [47]. Our pathway analyses identified TNF/NF-κB components among differentially methylated genes supporting a potential association with LDHC and SLC35G2 methylation [50,51]. For example, SLC35G2-dependent glycosylation could modulate FcεRI signaling, thereby plausibly influencing TNF/NF-κB activity [44,48]. Similarly, LDHC’s role in lactate metabolism may modulate immune cell activation thresholds, aligning with inflammatory processes [44]. Yao demonstrated that PPAR-γ activation suppresses TNF/NF-κB, decreasing mast cell degranulation [50]. Nevertheless, the specific connection between LDHC/SLC35G2 methylation and TNF/NF-κB signaling remains a testable hypothesis and requires functional validation. Genes responding to systemic corticosteroids exhibited methylation modifications nearly three times greater than those associated with food allergy (*p* = 4.42 × 10^−14^), indicating overlap with allergy-related epigenetic modifications [42]. However, the cross-sectional nature of methylation data limits definitive causal interpretation [42].

### 4.3. Research Design Advantages and Constraints

Our findings were corroborated through analysis of supplementary GEO dataset GSE114135, originating from the same investigative team, which afforded within-cohort validation while falling short of complete independent verification. This reliance on datasets derived from identical research groups and demographic populations constrains the generalizability of our conclusions, necessitating broader confirmation across diverse independent cohorts. Machine learning algorithms demonstrated robust performance metrics (AUC exceeding 0.85; referenced in Figure 9), with comprehensive ROC/precision-recall and calibration evaluations detailed in the Section 3. To mitigate overfitting risks inherent in high-dimensional methylation datasets, we implemented 10-fold cross-validation protocols, hyperparameter tuning procedures, and stacked autoencoder architecture for feature dimensionality reduction. Nevertheless, these methodological safeguards may inadequately address overfitting concerns given the substantial dimensionality of methylation profiles relative to our constrained sample populations, with observed inconsistencies in data partitioning suggesting additional optimization requirements. Additionally, insufficient documentation of preprocessing methodologies, encompassing normalization strategies and batch effect mitigation, potentially introduces analytical variability that subsequent investigations should resolve through comprehensive methodological transparency. Although cell-type deconvolution procedures were executed, residual cellular heterogeneity may continue to confound methylation signatures, emphasizing the necessity for more exhaustive analytical approaches. Prospective research endeavors should incorporate nested cross-validation frameworks, permutation-based statistical testing, expanded independent cohort recruitment, and thoroughly documented preprocessing protocols to enhance analytical reliability and experimental reproducibility.

In the initial analyses, class imbalance led to biased results in some algorithms. To mitigate this issue, stratified sampling was applied within each cross-validation fold to maintain the proportional representation of the classes. This approach enabled fairer evaluation of the models and reduced performance discrepancies between classes. Nevertheless, future studies may benefit from exploring oversampling, undersampling, or cost-sensitive learning methods to further enhance balanced performance. In addressing class imbalance, we initially tested additional resampling strategies, including oversampling and undersampling techniques. However, these approaches did not yield performance improvements and, in certain cases, even degraded classification accuracy, as reflected in the confusion matrix results. By contrast, stratified sampling consistently provided the most balanced and stable performance across classifiers. Therefore, to avoid introducing artificial variability or excessive data reduction, stratified sampling was adopted as the final approach in this study. This methodological choice, supported by supplementary confusion matrix analyses, highlights stratification as the most reliable strategy for mitigating imbalance-related misclassification in our dataset.

### 4.4. Clinical Application and Future Research

LDHC and SLC35G2 methylation profiles show promise as biomarkers for food allergy diagnosis and for predicting treatment response. Their use in early, non-invasive diagnosis could enhance patient outcomes by identifying at-risk individuals, including those with atopic histories, before severe allergic reactions develop. For instance, methylation-based tests could be developed to screen high-risk groups, such as children with suspected food allergies, or to monitor disease development in clinical practice. These biomarkers may also inform personalized treatment approaches, including customizing oral immunotherapy or evaluating corticosteroid responsiveness, by linking methylation patterns to clinical outcomes such as symptom severity or allergen-specific IgE concentrations.

Key limitations include reliance on datasets from a single research group and limited sample sizes. Future research should emphasize: (i) multi-center cohorts with varied populations, including pediatric and adult patients, to improve generalizability, (ii) transparent documentation of preprocessing methods, including normalization and batch effect correction, to ensure reproducibility, (iii) robust validation approaches such as nested cross-validation and permutation testing to address overfitting, (iv) additional molecular validation, including targeted bisulfite sequencing, to confirm methylation patterns, and (v) longitudinal studies to evaluate methylation stability and its relationship with disease progression or recovery. These measures will enhance the clinical value of LDHC and SLC35G2 as actionable biomarkers for food allergy management.

## 5. Conclusions

In conclusion, this study highlights the critical need for improved diagnostic accuracy in distinguishing individuals with food allergies from those with food sensitivity, especially considering the limitations of current diagnostic methods. The research introduces an innovative machine learning methodology that incorporates DNA methylation (DNAm) data, aiming to enable accurate diagnosis of food allergies and potentially uncover associated epigenetic targets. The integration of machine learning with epigenetic patterns holds significant potential for advancing disease prediction and enabling the discovery of previously unknown features within the epigenome.

To identify influential genes in the context of food allergies, our hybrid models, combining dimensionality reduction with various machine learning techniques, underwent rigorous experimental validation. The utilization of DMPS methods, combined with advanced algorithms such as SVM-poly, SVM-rbf, k-NN, Random Forest, and ANN, formed the basis of our analysis. Additionally, the integration of deep learning via stacked autoencoder (SAE) contributed to a robust and comprehensive analytical framework. To the best of our knowledge, this study is the first to demonstrate the impact of LDHC and SLC35G2 methylation on food allergy, positioning these genes as novel and promising epigenetic biomarkers.

This research serves as a cornerstone for advancing precision medicine in food allergy diagnosis, highlighting the potential of computational approaches to unravel the complex mechanisms of epigenetic regulation in disease pathways. Notably, the methylation pattern of the LDHC gene emerged as a potential biomarker for predicting food allergies using DNA methylation in this extensive analysis. Future work should prioritize the development of clinical tests that can accurately distinguish individuals with food allergies from those who are merely sensitized, thereby reducing the risk of misdiagnosis. A thorough examination of the identified genes and pathways is crucial for uncovering the complex mechanisms behind food allergies and exploring potential therapeutic options. Advances in bioinformatics tools will contribute to a better understanding of food allergies at the epigenetic level, paving the way for innovative diagnostic and treatment approaches.

## Figures and Tables

**Table 1:** Optimized hyperparameters of machine learning and deep learning methods (determined via grid search).

Algorithm	Hyperparameter	Value
**SVM—Polynomial**	C (Regularization)	1.0
	Kernel	Polynomial
	Degree	3
	Gamma	‘scale’
**SVM—RBF**	C (Regularization)	1.0
	Kernel	RBF
	Gamma	‘scale’
**Random Forest**	n_estimators	100
	max_depth	None
	min_samples_split	2
	min_samples_leaf	1
	max_features	‘sqrt’
**Neural Network**	Hidden layers	1 (64 neurons)
	Activation (hidden)	ReLU
	Activation (output)	Sigmoid
	Optimizer	Adam
	Learning rate	0.001
	Batch size	32
**k-NN**	n_neighbors	5
	Weights	Uniform
	Metric	Euclidean (Minkowski, *p* = 2)
**Stacked Autoencoder (SAE)**	Layers	3 (100–150–200 neurons)
	Activation	ReLU
	Regularization	L2 = 0.001
	Sparsity parameter	1

**Table 2:** Mean results of the accuracy obtained by the classification algorithms from the original dataset with DMPs feature selection.

Method	Accuracy	Precision	Recall	F-Score
SVM-Poly	0.7630	0.7806	0.7706	0.7770
SVM-RBF	0.8035	0.8064	0.8064	0.8064
KNN	0.6205	0.6153	0.6236	0.6214
Random Forest	0.8356	0.8403	0.8403	0.8403
ANN	0.8374	0.8396	0.8445	0.8405

**Table 3:** Mean results of the accuracy obtained by the classification algorithms from the original dataset with SAE feature selection.

Method	Accuracy	Precision	Recall	F-Score
SVM-Poly	0.8402	0.8601	0.8010	0.8380
SVM-RBF	0.8192	0.8381	0.8245	0.8336
Decision tree	0.8713	0.8710	0.8710	0.8710
Random Forest	0.8914	0.9010	0.8610	0.8700
ANN	0.8780	0.8515	0.9008	0.8698

**Table 4:** The list of genes obtained as a result of the experimental evaluations.

Gene Symbol	Location	Pearson Correlation	*p* Value
**TNF**	body	0.13	9.49 × 10^−3^
**RGS12**	body	0.132	8.76 × 10^−3^
**FRA10AC1**	promoter	−0.137	6.27 × 10^−3^
**MAD1L1**	body	−0.123	3.32 × 10^−3^
**RP4-735C1.4**	body	−0.255	2.85 × 10^−3^
**CTD-2535L24.2**	body	−0.169	2.13 × 10^−3^
**CSRP1**	body	−0.22	1.86 × 10^−3^
**HCG4B**	promoter	−0.176	1.41 × 10^−3^
**CBR1**	body	−0.277	1.16 × 10^−3^
**GSTM3**	body	−0.278	1.10 × 10^−3^
**AP000688.14**	body	−0.197	3.40 × 10^−4^
**ZNF267**	body	−0.235	3.40 × 10^−4^
**RP4-583P15.15**	body	−0.147	1.72 × 10^−4^
**LIME1**	body	−0.208	1.50 × 10^−4^
**HLA-K**	body	−0.221	5.58 × 10^−5^
**LDHC**	promoter	−0.578	7.60 × 10^−22^
**CLECL1**	body	−0.411	7.17 × 10^−7^
**AXIN2**	body	−0.305	1.78 × 10^−7^
**SLC35G2**	promoter	0.598	1.81 × 10^−14^
**SETD4**	body	0.316	1.28 × 10^−14^
**RP11-134G8.7**	body	−0.442	4.74 × 10^−17^

**Table 5:** Functional enrichments analyses of DNA methylated genes in food allergy.

Biological Process (Gene Ontology)	Strength	False Discovery Rate
Regulation of calcidiol 1- monooxygenase activity	2.54	0.0337
Positive regulation of I-kappaB phosphorylation	2.47	0.0381
Cellular response to nicotine	2.47	0.0381
Regulation of vitamin D biosynthetic process	2.47	0.0381
Necroptotic signaling pathway	2.47	0.0381
**Cellular Component** **(Gene Ontology)**	**Strength**	**False** **Discovery Rate**
Tumor necrosis factor receptor superfamily complex	2.71	0.0269
**Local Network Cluster** **(STRING)**	**Strength**	**False** **Discovery Rate**
Beta-catenin destruction complex	2.54	0.0112
Defective RIPK1-mediated regulated necrosis and TRAF-type zinc finger	2.54	0.0112
TNFR1-induced signaling pathway	2.35	0.00026
**KEGG Pathways**	**Strength**	**False** **Discovery Rate**
Antifolate resistance	1.83	0.0049
Adipocytokine signaling pathway	1.79	4.08 × 10^−5^
RIG-I-like receptor signaling pathway	1.78	4.08 × 10^−5^
NF-kappa B signaling pathway	1.71	1.50 × 10^−5^
TNF signaling pathway	1.67	1.50 × 10^−5^
**Reactome Pathways**	**Strength**	**False** **Discovery Rate**
TNFR1-mediated ceramide production	2.54	0.0093
Defective RIPK1-mediated regulated necrosis	2.47	0.01
Dimerization of procaspase-8	2.28	0.02
CASP8 activity is inhibited	2.28	0.02
Regulation by c-FLIP	2.28	0.02

**Table 6:** Classification performance of machine learning models evaluated with 10-fold cross-validation on the external validation cohort (GSE114135).

Method	Accuracy	Precision	Recall	F-Score
Random Forest	0.830	0.85	0.82	0.805
SVM-RBF	0.820	0.83	0.81	0.790
ANN	0.800	0.81	0.79	0.800
SVM-Poly	0.785	0.80	0.77	0.785
k-NN	0.750	0.74	0.72	0.735

## Data Availability

The datasets generated during and/or analyzed during the current study are available in the GEO database repository, [https://www.ncbi.nlm.nih.gov/geo/query/acc.cgi?acc=GSE114134] (accessed on 10 July 2025).
